# Alternative treatment option for high risk surgical candidates after previous cardiac surgery: The Scottish National TAVI Programme experience

**DOI:** 10.1186/1749-8090-10-S1-A236

**Published:** 2015-12-16

**Authors:** Vincenzo Giordano, Ashok Kar, Andrew Flapan, Neil Uren, Sai Prasad, Renzo Pessotto

**Affiliations:** 1 Department of Cardiothoracic Surgery, Royal Infirmary of Edinburgh, Edinburgh, EH16 4SA, UK; 2 Department of Cardiology Royal Infirmary of Edinburgh, Edinburgh, EH16 4SA, UK

## Background/Introduction

Patients that require aortic valve replacement, having previously undergone cardiac surgery, have a higher morbidity and mortality with increased risks of mediastinal injury during re-operation.

## Aims/Objectives

In the era of TAVI for high risk surgical candidates, we analysed our experience with redo conventional surgical aortic valve replacement (SAVR) and compared these results with those from patients who had undergone TAVI as a second heart operation.

## Method

A retrospective, observational, comparative study was performed in the national centre offering TAVI. 149 consecutive patients underwent redo operation with SAVR (n = 59) or TAVI (n = 90) between October 2012 and February 2015. In the SAVR group patients with concomitant procedures (n = 8) or endocarditis (n = 8) were excluded from the analysis.

## Results

Mean logistic Euroscore was 29.6 for redo-TAVI compared to 18.6 for redo-SAVR (p < 0.05). Type of previous cardiac surgery performed in each redo group is summarised in Table 1.

**Table 1:** 

Previous Cardiac Surgery	Redo-SAVR	Redo-TAVI
CABG	20	82
AVR	28	2
AVR + CABG	1	0
MVR	6	4
Congenital	4	0
Other Cardiac	0	2

TAVI access was transfemoral (65.5%), transapical (6.7%), or trans-aortic (27.8%).

Despite a higher incidence of MACE (major adverse cardiac events) in SAVR patients, results indicated no significant differences between two groups in terms of all-cause and cardiovascular related mortality, stroke, myocardial infarction.

In details, hospital mortality was higher in SAVR group (5.1%) compared to TAVI group (2.2%), although it did not reach statistical significance.

There was a higher incidence of CVA in the SAVR versus TAVI group (6.8% vs. 2.2%; p = 0.385).

Patients were found to require permanent pacemaker insertion more often after TAVI (10% vs 3.4%; p = 0.2).

Finally, average length of post-operative hospital stay was significantly shorter in TAVI than SAVR group (5.4 vs 10.4 days ; p < 0.00001).

## Discussion/Conclusion

This retrospective analysis suggests that TAVI is preferable to SAVR in this high risk group of patients. Redo-SAVR remains a valid option for younger patients in whom long-term durability of the prosthetic valve is a concern.

